# Acute Coronary Syndrome, Antiplatelet Therapy, and Bleeding: A Clinical Perspective

**DOI:** 10.3390/jcm9072064

**Published:** 2020-07-01

**Authors:** Gregorio Tersalvi, Luigi Biasco, Giacomo Maria Cioffi, Giovanni Pedrazzini

**Affiliations:** 1 Division of Cardiology, Fondazione Cardiocentro Ticino, 6900 Lugano, Switzerland; giacomomaria.cioffi@bluewin.ch; 2 Department of Internal Medicine, Hirslanden Klinik St. Anna, 6006 Lucerne, Switzerland; 3 Azienda Sanitaria Locale Torino 4, Ospedale di Ciriè, 10073 Ciriè, Italy; luigi.biasco@gmail.com; 4 Department of Biomedical Sciences, University of Italian Switzerland, 6900 Lugano, Switzerland; 5 Department of Cardiology, Kantonsspital Luzern, 6000 Lucerne, Switzerland

**Keywords:** dual antiplatelet therapy, bleeding, acute coronary syndrome, high bleeding risk, clopidogrel, prasugrel, ticagrelor, tailored therapy, P2Y12 inhibition

## Abstract

Inhibition of platelet function by means of dual antiplatelet therapy (DAPT) is the cornerstone of treatment of acute coronary syndrome (ACS). While preventing ischemic recurrences, inhibition of platelet function is clearly associated with an increased bleeding risk, a feared complication that may lead to significant morbidity and mortality. Since bleeding risk management is intrinsically associated with therapeutic adjustments undertaken during the whole clinical history of patients with acute coronary syndrome, single decisions taken from the very first day to years of follow-up might be decisive. This review aims at providing a clinically oriented, patient-tailored approach in reducing the risk and manage bleeding complications in ACS patients treated with DAPT. The steps in clinical decision making from the day of ACS to follow-up are analyzed. New treatment strategies to enhance the safety of DAPT are also described.

## 1. Introduction

Inhibition of platelet function following acute coronary syndrome (ACS) using dual antiplatelet therapy (DAPT) is aimed at preventing the occurrence of short- and long-term thrombotic complications.

In the first weeks after percutaneous revascularization DAPT reduces the risk of stent thrombosis, a potentially fatal event occurring as a result of inflammation and endothelial damage associated with mechanical insult during percutaneous coronary interventions (PCI), the so-called “stent protective effect” [1,2]. As time passes by, the aim of DAPT changes. Long-term therapy has been shown to mitigate the risk of subsequent ischemic events not only associated with culprit lesions/vessels, but also arising from the progression of atherosclerosis, also known as the “patient protective effect” [3,4]. 

Several antithrombotic drugs have been proposed over time aimed at providing the highest thrombotic protection while counterbalancing the associated hemorrhagic risks. According to European guidelines [5,6,7] the use of the two most recent and potent P2Y12 inhibitors (i.e., prasugrel, ticagrelor) on top of aspirin with or without PCI is nowadays recommended in all suitable ACS patients [8,9]. A greater reduction of the thrombotic risk comes at the price of an increase in major bleedings, occurring in around 1–8% during the first year after DAPT initiation [8,9,10,11,12,13]. Even less severe bleeding might increase mortality via indirect mechanisms such as unplanned hospitalization, need for urgent procedures, and DAPT discontinuation [14]. As a result, bleeding is directly associated with increased mortality and indirectly linked to recurrence of ischemic events such as myocardial infarction (MI) and stroke [15,16,17,18,19,20]. 

Even in the presence of clear and precise guidelines and dedicated scores, a wise choice of type and length of DAPT therapy is challenging. Overall, DAPT should be tailored over time on the basis of the clinical profile, the type of intervention, patient’s tolerance, comorbidities, and occurrence of bleedings events [21]. Tailoring antiplatelet therapy intensity to patient risk improves not only clinical outcomes, such as morbidity and mortality, but it also impacts on health utility and could produce cost savings [22].

**Figure 1:**
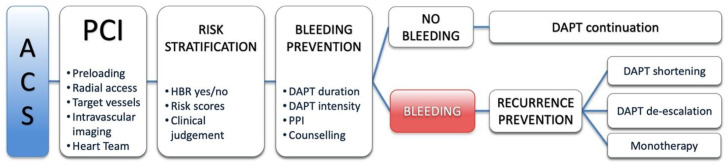
Critical points in decision making when choosing an antiplatelet strategy and managing related bleedings. PCI, percutaneous coronary intervention; DAPT, dual antiplatelet therapy; ACS, acute coronary syndrome; HBR, high bleeding risk; PPI, proton pump inhibitors.

The purpose of this review is to offer an update on current literature and provide a clinically oriented, patient-tailored approach in reducing the risk and manage bleeding complications in ACS patients treated with DAPT (Figure 1).

## 2. Definition of Bleeding and Prognostic Significance

The definition and classification of bleeding severity has always suffered from extensive variability with several classifications and study-specific definitions proposed over time [23], making interstudy comparisons challenging. To limit the degree of heterogeneity, the Bleeding Academic Research Consortium (BARC) proposed a classification system which has become widely accepted as a common framework for reporting bleedings (Table 1) [24]. More recently, a consensus definition to identify patients at high bleeding risk (HBR) was proposed, a peculiar subgroup either excluded or underrepresented in clinical trials [25].

The negative prognostic impact of bleeding has been reported in a series of large clinical trials in ACS patients. The mechanisms linking bleeding to increased mortality are heterogeneous [26]. While intracranial or massive bleeding exerts a direct life threatening effect due to fatal brain damage or sudden cardiocirculatory collapse, other forms of less severe bleeding increase the risk of death via indirect mechanisms. The need for transfusions of red blood cells, by increasing inflammation and apoptosis, is a possible link between bleeding and mortality [27]. Other medical procedures required to manage bleeding may also increase the rates of complications. Furthermore, bleeding is a major driver of unplanned DAPT discontinuation, which increases the risk of ischemic recurrences [28]. Besides DAPT, other key cardioprotective medications like beta-blockers, Renin–Angiotensin–Aldosterone System (RAAS) blockers or statins are often discontinued after bleeding, further increasing the risk for recurrent events [29]. 

Reflecting the importance of the prognostic association between bleeding and mortality, current guideline recommendations advocate for bleeding avoidance strategies starting from the time of revascularization and continuing in the long-term after the acute clinical presentation [7].

## 3. Risk Stratification

Quantifying patients’ bleeding risk through the assessment of their clinical characteristics is crucial as it determines the type and length of DAPT regimen. Recently, the Academic Research Consortium for High Bleeding Risk (ARC-HBR) defined HBR as risk of BARC 3 or 5 bleeding ≥4% or of intracranial hemorrhage ≥1% at 1 year [25]. Clinical criteria identifying HBR are older age, kidney and/or liver disease, active cancer, anemia, low platelet count, previous stroke, prior bleeding, recent trauma or surgery, and use of oral anticoagulants and/or NSAIDs [7,25,30,31,32]. By accounting for these variables, the ACR-HBR score allows a dichotomic stratification of patients in HBR and non-HBR [25]. Several other scores have been proposed, a summary of their key points is provided in Table 2. Importantly, many variables coincide as risk factors for bleeding as well as ischemic events (e.g., older age, male gender, high creatinine, low hemoglobin) [33]. As a result, physicians must often grapple with patients at high risk with a narrow therapeutic window in both ways.

European guidelines gave an intermediate grade recommendation for the use of risk scores such as the DAPT and the PRECISE-DAPT (class IIb recommendation, level of evidence A) to guide antiplatelet therapy after PCI [7]. However, none of these risk prediction models have been prospectively tested in the setting of randomized controlled trials (RCT). In our clinical practice, scores do not add valuable information in addition to clinical judgement when all above parameters are taken into consideration. Thus, when assessing the risk, clinical judgment should be carefully exercised. 

## 4. Bleeding Prevention in Different DAPT Phases

Bleeding risk management is intrinsically associated with therapeutic choices taken during the whole clinical history of patients with ACS. Thus, single decisions taken from the very first day to years of follow-up after an ACS might be decisive. 

### 4.1. Pre-Treatment

In invasively managed ACS, pre-treatment refers to the practice of initiating DAPT before invasive coronary angiography and percutaneous treatment [40]. 

The rationale of P2Y12 receptor blocker administration before PCI in ACS arises from the observation that the risk of early thrombotic complications, such as re-infarction or acute stent thrombosis, is directly related to the level of platelet reactivity. This clearly represents an attractive option when either an emergent or urgent/elective approach is undertaken. Nonetheless, exposure to a more potent platelet inhibition might be useless in patients in whom an ACS is ruled out after angiography (e.g., myocarditis, tako-tsubo cardiomyopathy, non-ischemic myocardial injury) and possibly harmful in of those referred to surgical revascularization, a small but clinically challenging subgroup of ACS patients [41]. In addition, pre-treatment often defines the type of P2Y12 inhibitor that will be prescribed to patients even after discharge, highlighting the importance of a wise choice in forecast of a long-term strategy.

In ST-elevation myocardial infarction (STEMI), there is limited evidence with respect to when the P2Y12 inhibitor should be initiated [42,43,44,45,46]. European guidelines suggest that the earliest possible administration may be preferable (not compulsory) to achieve rapid platelet’s inhibition. However, in cases in which the STEMI diagnosis is not clear, delaying P2Y12 inhibitor administration until coronary anatomy is known should be considered [6]. In non-ST-elevation myocardial infarction (NSTEMI), European guidelines discourage pre-treatment with Prasugrel [5,47,48]. Regarding the other two P2Y12 inhibitors, recent data from SCAAR (Swedish Coronary Angiography and Angioplasty Registry) suggested no benefit of a pre-treatment strategy with ticagrelor or clopidogrel on 30-day and 1-year mortality while highlighting a significant increase in bleedings [49]. 

In summary, the role of pre-treatment with P2Y12 inhibitors in ACS is still controversial. It might increase bleeding risk without giving major benefits on short- and long-term clinical outcomes.

Concordant to European Society of Cardiology (ESC) guidelines, a wise approach might be to perform routine pre-treatment in STEMI, except for patients with unclear diagnosis or high/very high bleeding risk, and to wait to administer the loading dose of P2Y12 inhibitor in NSTEMI only after defining coronary anatomy. 

### 4.2. Percutaneous Coronary Interventions

In patients with ACS undergoing PCI, a wise selection of procedural strategy might significantly reduce the short- and long-term bleeding risk.

The transition from the femoral to the radial access has dramatically reduced the risk of access site major bleedings, surgical access site repair, transfusion of blood products, death, MI, or stroke [50,51] while maintaining the level of intraprocedural anticoagulation and platelet inhibition substantially stable. Thus in all PCIs the radial artery should be considered the access of choice, and, given its advantages, interest is increasing in shifting from the femoral to the radial as an ancillary access in structural interventions [52]. 

Even the procedural strategy might indirectly impact on the long-term bleeding risk. Extensive percutaneous revascularizations in multivessel disease (or in prior failures of PCIs with need of stent in stent implantation), adoption of complex two stents techniques in particular when tackling left main lesions, treatment of calcific lesions with associated stent under-expansion, malapposition and small vessel disease are all associated with an increased risk of long-term ischemic events. Interestingly, those challenging anatomical characteristics are often recognized in elderly patients with associated comorbidities such as diabetes, advanced vascular disease or renal impairment, a group with an intrinsically associated HBR [30]. Procedural strategies might then aim at performing percutaneous revascularization targeting only those segments with a clear prognostic and functional relevance, choosing second and third generation coronary stents approved for short-term DAPT and optimizing the result of stent implantation with the support of intravascular imaging. In our routine, doubtful cases undergo Heart Team discussion to verify whether surgery might provide a more complete revascularization and a lower long-term exposure to bleeding risk at a reasonable surgical risk.

### 4.3. Stent Protection (Short-Term)

Following the guideline-recommended treatment interval of 12 months in ACS [7], much debate remains around the optimal antiplatelet therapy regimen and duration. 

During the first weeks after stent implantation, DAPT reduces the risk of stent thrombosis that occurs as a result of inflammation following the mechanical insult of PCI [1,2]. After this, DAPT has been shown to mitigate the risk of recurrent ischemic events associated with progression of coronary artery disease even beyond culprit-related lesions [3,4]. While DAPT does not limit the progression of atherosclerosis, platelet inhibition protects against consequences of spontaneous coronary plaque erosion or rupture [53]. Whereas a prolongation of DAPT beyond 1 year would therefore seem reasonable to reduce ischemic risk, continued antiplatelet therapy prolongs exposure to the associated bleeding risk [54,55]. Thus, potential benefits of reducing DAPT duration were evaluated in recent large RCTs (Table 3). Beside length of exposure, different DAPT regimens might impact on the associated bleeding risk. Pushing the boundaries of platelet inhibition to prevent ischemic events with more potent P2Y12 inhibitors is clearly associated to an increase in non-related to coronary artery bypass grafting major bleeding as observed in both PLATO [9] and TRITON-TIMI 38 [8] trials where prasugrel and ticagrelor where compared to clopidogrel, respectively. The only two large trials performing a direct comparison of prasugrel-based and ticagrelor-based DAPT in ACS patients did not show any difference in bleeding events between the two DAPT regimens [56,57].

### 4.4. Patient Protection (Long-Term)

In the PEGASUS-TIMI 54 trial [58], a prolonged DAPT with ticagrelor (90 mg twice daily or 60 mg twice daily) and low-dose aspirin 1 to 3 years after MI showed a significant reduction in cardiovascular death, MI, or stroke compared to aspirin and placebo on a median follow-up of 33 months. As a drawback, the rates of major bleeding were higher with ticagrelor (2.60% with 90 mg and 2.30% with 60 mg) than with placebo (1.06%; *p* < 0.001 for each dose vs. placebo). Similar results were obtained in the DAPT trial [4], in which continuation of DAPT with clopidogrel or prasugrel for more than 12 months after coronary stenting showed a reduction in non-fatal ischemic events, but an increase in major bleeding compared to single antiplatelet therapy (SAPT) with aspirin and placebo.

Current evidence suggests that bleeding risk is proportionally related to the DAPT duration both within and beyond 1 year of treatment duration. Extension of DAPT should therefore be individualized based on patients’ ischemic risk and should be avoided when the risk of bleeding overshadows the likelihood of ischemic recurrences. A recent retrospective study of pooled data of RCTs showed that patients who were treated with complex PCI had a higher risk of ischemic events but benefitted from long-term DAPT only if HBR features were not present. These data suggest that, when bleeding and ischemic risk are concordant, the first should guide decision-making on DAPT duration [59]. Thus, in patients presenting with ACS and at HBR *a priori*, a strategy of short DAPT may be an effective solution.

## 5. Management of Bleeding during DAPT

### 5.1. General Approach

DAPT-associated bleeding represents a clinical challenge in a field where no clear evidence is available. Overall, concurrent antiplatelet therapy is considered an important predictor of mortality and complications following a gastrointestinal, intracranial, or trauma-related bleeding [63,64,65,66].

Table 4 shows a stepwise approach the management of DAPT related bleedings according to current European guidelines [7].

Decisions regarding the acute therapy and whether to stop or continue DAPT are made on an individual patient basis, based primarily on the severity of bleeding, as this is the major predictor of morbidity and mortality [20,67]. A clear need to balance likelihood and consequences of therapy discontinuation according to the degree of the hemorrhagic event also has to be considered.

In the acute setting, patients presenting trivial or mild bleeding do not require specific therapy, but may profit from adequate drug-adherence counselling, as well as adding a PPI in cases of gastrointestinal (GI) bleeding. If the bleeding is moderate, its cause should be identified and treated (e.g., peptic ulcer, hemorrhoidal plexus, epistaxis, neoplasm). For severe and life-threatening bleeding, an urgent treatment of its source must be obtained together with red blood cells and platelet transfusions [7].

### 5.2. Gastrointestinal Bleeding

The most common serious DAPT related bleeding complication after PCI is GI haemorrage [68,69]. 

Aspirin causes GI bleeding due to its direct inhibition of Cyclooxygenase-1, thus reducing the endothelial protective effect of prostaglandins. P2Y12 inhibitors are believed not to be directly ulcerogenic, but to impair ulcer healing by blocking platelet aggregation, angiogenesis, and endothelial proliferation [70]. Ticagrelor and prasugrel have been associated with higher risk of GI bleeding compared to clopidogrel [8,69]. 

GI bleeding in patients with recent ACS and/or PCI represents a great therapeutic challenge due to its insidious presentation. The need to attain hemostasis often requires the premature discontinuation of antithrombotic therapies. In addition, acute bleeding itself leads to platelet activation and creation of a prothrombotic *milieu*. These two factors may explain the increase in ischemic risk in patients with GI bleeding receiving DAPT after ACS [71]. 

Often, GI bleeding might be only suspected in a patient with a progressive hemoglobin drop without overt bleeding source. Endoscopy is often crucial, after lesion identification, epinephrine injection, sclerotherapy, and metal clip placement [72] can effectively solve bleeding. In emergent cases, endovascular angiography is an effective and safe alternative to surgical intervention for patients whose GI bleeding is refractory to medical and endoscopic treatment [73] When GI bleeding is caused by diffuse lesions or super-selective catheterization is not possible, vasopressin infusion may be the only remaining therapeutic option, in particular in low GI bleedings [74] with a success rate of 59–90% [75]. Efficacy of octreotide in the prevention of recurrent bleeding from gastrointestinal angiodysplasia has been also reported and is currently used in practice [76].

If endoscopy shows no active bleeding in patients with suspected upper GI bleeding, continuing DAPT is recommended [7,77], while a short interruption of aspirin (three days) should be considered [7,77], (preferably with continuation of P2Y12 inhibitor if feasible) when an active source is found. In overt lower GI bleeding, P2Y12 can be stopped for a maximum of 7 days, whilst aspirin should be continued [78]. Importantly, in patients with a recent (<90 days) ACS or percutaneous intervention (<30 days), DAPT should be continued [78]. While not often specified by current guidelines, DAPT discontinuation should be preferentially considered in setting with 24/7 cath lab facility availability to timely treat potential recurrent ischemic events, in particular in patients with recent (<30 days) PCI. 

To reduce the risk of further GI bleeding, pharmacologically limiting the gastric acid secretion with the use of proton pump inhibitors (PPI) has been demonstrated to exert a protective effect [79]. Based on the assumption that the benefit of PPI prescription outweighs its harm, the 2017 ESC focused update on DAPT recommends prescription of PPI therapy to all patients receiving DAPT [7].

### 5.3. Intracranial Bleeding

Intracranial bleeding (ICB) represents the most serious DAPT-associated adverse event. Different locations of ICB are associated with different recurrent rates and disabilities. ICB are classified as lobar (cerebral cortex and underlying white matter) or deep (basal ganglia, thalamus, brainstem), with recurrence rates of 15.7% and 3.4%, respectively [80]. In two recent prospective observational studies on patients with ICB, antiplatelet therapy on admission was associated with a higher 24-h [65], in-hospital [64], and 3-month [65] mortality rate compared to naïve patients.

In the acute setting, patients with ICB should be monitored and managed in an intensive care unit or dedicated stroke unit with high expertise level. All anticoagulant and antiplatelet drugs should be discontinued. While anticoagulation should be antagonized with proper agents, the specific management of patients on DAPT remains unclear, since platelet transfusions have shown inferiority to standard care regarding mortality [81]. If rupture of an intracranial aneurysm is evident, an interventional strategy with endovascular coiling has to be taken into consideration [82].

Enormous difficulties as to whether and when to restart medication are clearly evident in survivors [83]. In the large trials comparing new P2Y12 inhibitors to Clopidogrel, ICB was reported in only 0.3% of patients [8,9]. A meta-analysis including six observational studies on patients with ICB while on SAPT showed that only 43% of patients resumed antiplatelet therapy, and the timing of resumption, when available, varied widely from weeks to months [84]. 

Due to the scarce number of ICB events in DAPT trials, we can only presume that DAPT might be reinitiated after approximately 4 weeks, with aspirin plus clopidogrel, and continued until the minimum advised duration [5,83]. Clearly, reassessment of clinical status, need for DAPT (time from ischemic event/revascularization), and team discussion with neurologist/radiologist/cardiologist should guide the decision making process.

## 6. Treatment Strategies after Bleeding

### 6.1. Restarting Antiplatelet Therapy 

The need for antiplatelet therapy must always be re-evaluated after a bleeding event and tailored on the patient’s risk of recurrent ischemic and hemorrhagic events. Importantly, the risk of new ischemic events is increased after a recent ACS, especially during the first 3 months, and remains elevated up to 1 year [14]. After bleeding, no RCTs have assessed whether stopping or restarting one or both antiplatelet agents is the best choice. In patients with a recently implanted coronary stent, premature discontinuation of one or both antiplatelet agents (especially the P2Y12 inhibitor) has been shown to be the strongest predictor of stent thrombosis [85,86,87].

Recovery of platelet function after P2Y12 discontinuation depends on the magnitude of on-treatment platelet inhibition. On average, platelet reactivity returns to baseline by washout day 7 after prasugrel, day 5 after clopidogrel, and day 4–5 after ticagrelor [88,89,90]. Thus, the risk of stent thrombosis increases with longer time off treatment, particularly more than 5 days, and if treatment is stopped within the first month after the procedure [85,86,91,92].

In a contemporary registry of over 5000 patients treated with PCI, cardiovascular risk was significantly increased when DAPT cessation was due to non-compliance or bleeding [93].

The risk was highest for the first 7 days after discontinuation of DAPT, but still high within 30 days [93]. In a recent analysis of the PARIS registry, DAPT discontinuation due to bleeding occurred in 5% of the patients and it had higher subsequent mortality [28]. 

In summary, early discontinuation of DAPT is inevitably correlated to ischemic events and mortality. Thus, efforts should be made to continue antiplatelet therapy unless the bleeding is considered severe [7], this being the only case that justifies early DAPT interruption. Timing of DAPT resumption after clinically significant bleeding varied widely in clinical trials, from 2–3 days in GI bleeding to months after ICB [84]. Indications and timing of DAPT resumption should be evaluated on a patient basis, often achieving a consensus by multidisciplinary consensus. Due to the high ischemic risk already in the first days after interruption, DAPT should be discontinued under strict medical surveillance and restarted as soon as deemed safe. However, if the standard post-ACS DAPT with aspirin and a potent P2Y12 is considered hazardous, other strategies of optimization of antithrombotic therapy after a bleeding event are possible, which should be tailored on the individual patient’s risk.

### 6.2. Preventing Bleeding Recurrence

Several therapeutic possibilities have been proposed to minimize the risk of bleeding in patients on DAPT. Three different strategies to modulate antiplatelet therapy that have been tested in clinical trials.

#### 6.2.1. DAPT Shortening

Since bleeding risk is proportionally related to DAPT duration, reducing exposure to antiplatelet therapy has been hypothesized as a valid strategy to reduce bleedings. 

Recent evidence has shown net benefits in the ischemic/bleeding balance for short (≤6 months) vs. 12 months DAPT in patients with low ischemic risk [94,95]. Seven major RCTs published in recent years and evaluating a very short DAPT (1 or 3 months) after stenting as compared to 12-month therapy [60,61,96,97,98,99,100] were analyzed together in a recent metanalysis [101]. Interestingly, very short DAPT yielded comparable rates of all-cause mortality, stent thrombosis, and major acute cardiovascular endpoints than 12 months DAPT. At the same time, very short DAPT was associated with reduced rates of major bleeding or any bleeding. Subgroup analyses showed consistent results for 1 vs. 3 month DAPT and for aspirin vs. P2Y12 inhibitor monotherapy following very short DAPT [101]. 

After a bleeding event, DAPT shortening is reasonable as these patients are mostly considered at high bleeding risk [38]. A 1-month DAPT regimen is considered safe after treatment with newer generation drug-eluting stents tested for 1-month DAPT [102].

#### 6.2.2. DAPT De-Escalation

If bleeding occurs in patients on DAPT with prasugrel or ticagrelor, its replacement with clopidogrel (“de-escalation”) may be another possible option. 

Two aspects are of particular importance when considering a DAPT de-escalation strategy. First, timing of de-escalation should be carefully evaluated. If de-escalation is performed too close to the index event, there is a potential risk of increasing the rate of ischemic complications because the first weeks after ACS represent a vulnerable time window with high risk for ischemic recurrences or stent thrombosis [23,103]. Supported by current evidence, de-escalation is considered safe at the earliest 1 month after ACS [104].

The second important aspect is the considerable rate of high platelet reactivity with clopidogrel, occurring in up to 30% of patients [105] and representing a possible threat for thrombotic complications. This high patient-to-patient variability in platelet response to clopidogrel is multifactorial, being only partially explained by individual factors such as body weight, diabetes, renal failure, and old age [106,107,108], and also implicating genetic variants of liver cytochromes [109].

Platelet function tests (PFT) may be used as optional tool for deciding on a de-escalation from prasugrel or ticagrelor to clopidogrel [110]. 

In summary, de-escalation of DAPT is a valid strategy after a bleeding of low or moderate entity, since it represents an intermediate step between maintaining DAPT with potent P2Y12 inhibitors and switching to SAPT. The use of PFT to evaluate the patient’s response to clopidogrel after switching might be considered as an additional tool to lower the risk of ischemic events. 

#### 6.2.3. Monotherapy with P2Y12 Inhibitors

The third strategy that has gained more and more evidence in the recent years is represented by the switch from DAPT to SAPT with P2Y12 inhibitors. In experimental models, the potent blockade of the P2Y12 receptor by prasugrel was able to attenuate the thromboxane A2-dependent pathways of platelet activation, which is the target of aspirin therapy [111]. Furthermore, in a recent RCT in 44 volunteers, ticagrelor-monotherapy and ticagrelor-based DAPT comparably affected hemostatic system activation [112]. These observations suggest that in the presence of potent P2Y12 blockade, adjunctive use of aspirin might have a limited impact.

Major RCTs evaluating P2Y12 monotherapy after short or very short DAPT and subsequent analyses of their results were described above. In general, P2Y12 monotherapy seems to reduce bleeding events without worsening of ischemic outcomes.

In the recent Ticagrelor With Aspirin or Alone in High-Risk Patients After Coronary Intervention (TWILIGHT) trial, ticagrelor monotherapy (following an initial course of uncomplicated DAPT for 3 months) reduced the rate of BARC 2, 3, or 5 bleeding compared with standard DAPT consisting of ticagrelor and aspirin for 12 months. Of note, the majority of patients (64%) included in the study had an ACS diagnosis at baseline. Anti-ischemic efficacy, evaluated using a composite endpoint of all-cause death, non-fatal MI, and stroke, did not differ among the randomized treatment groups, thus confirming the promising role of this regimen. 

Monotherapy with P2Y12 inhibitors is usually the best choice after a moderate or severe GI bleeding event, because it avoids the ulcerogenic effects of aspirin on the gastric mucosa that may reiterate the bleeding. In addition, P2Y12 monotherapy may also be a good strategy in patients with mild but frequent bleeding events, in order to avoid complete suspension of antiplatelet therapy which carries ominous consequences [28].

At present, large clinical trials are ongoing with the aim to further clarify the role of aspirin-free strategies in high-risk patients after ACS (ULTIMATE-DAPT (NCT03971500), STOPDAPT-2-ACS (NCT03462498)). Importantly, physicians should keep in mind that SAPT with P2Y12 has been tested only after at least 1 month of DAPT. 

#### 6.2.4. De-Escalating DAPT in Anticoagulated Patients

Almost 6–8% of patients undergoing PCI have an indication for chronic oral anticoagulation (OAC) with vitamin K antagonists (VKA) or direct oral anticoagulants (DOAC), as a result of various conditions such as atrial fibrillation, mechanical heart valves and recent or recurrent venous thromboembolism [7]. However, adding antiplatelet agents to OAC increases non-fatal and fatal bleeding risk more than 3-fold compared with DAPT [113,114].

Therefore, clinical judgment and regular reassessment of the indication for OAC is essential.

As a golden rule, clopidogrel should be the P2Y12 inhibitor of choice, and triple therapy (DAPT plus OAC) should be kept as short as possible. Other possible strategies to avoid bleeding complications in this group of patients are preferring DOACs instead of VKA, targeting INR 2–2.5 when VKA is used, using the lower DOAC regimen tested in approval studies, and routine use of PPI [7]. 

In the case of a bleeding event in patients with DAPT and OAC, we suggest the de-escalation to dual therapy with OAC and Clopidogrel. If the patient is already on dual therapy, consider discontinuing antiplatelet therapy if deemed safe.

## 7. Conclusions

Since bleeding events have a major impact on prognosis in patients on DAPT after ACS, physicians’ effort should be maximized to prevent this complication. This relies on a clinical and patient-tailored approach, that starts from the index hospitalization to the whole follow up. Accurate risk stratification, technical appropriateness of interventional procedures, wise choice of DAPT regimens, and duration are important steps in preventing bleedings event after ACS. 

When occurring, timely management and a proper strategy in secondary prevention of bleeding events are decisive factors to reduce morbidity and mortality in these patients.

Importantly, when bleeding and ischemic risk are concordant, bleeding risk should inform decision making on DAPT. 

## Figures and Tables

**Table 1:** Bleeding Academic Research Consortium definition for bleeding (modified from Mehran et al. [24]). CABG, coronary artery bypass grafting; IV, intravenous.

**Type 0**	No bleeding
**Type 1**	Bleeding that is not actionable and does not cause the patient to seek treatment
**Type 2**	Any clinically overt sign of hemorrhage that “is actionable” and requires diagnostic studies, hospitalization, or treatment by a health care professional
**Type 3**	a. Overt bleeding plus hemoglobin drop of 3 to <5 g/dL (provided hemoglobin drop is related to bleed); transfusion with overt bleeding b. Overt bleeding plus hemoglobin drop <5 g/dL (provided hemoglobin drop is related to bleed); cardiac tamponade; bleeding requiring surgical intervention for control; bleeding requiring IV vasoactive agents c. Intracranial hemorrhage confirmed by autopsy, imaging, or lumbar puncture; intraocular bleed compromising vision
**Type 4**	CABG-related bleeding within 48 h
**Type 5**	a. Probable fatal bleeding b. Definite fatal bleeding (overt or autopsy or imaging confirmation)

**Table 2:** Risk scores used to estimate bleeding risk in patients with coronary artery disease on antiplatelet therapy. * In the ACTION score, a lower body weight was associated to higher bleeding risk, with maximum risk when body weight was ≤50 kg. In the PARIS score, a body mass index <25 or ≥35 was associated to higher bleeding risk. ACS, acute coronary syndrome; ATT, antithrombotic treatment; BP, blood pressure; CAD, coronary artery disease; ECG, electrocardiogram; HF, heart failure; NSTEACS, non-ST-elevation acute coronary syndrome; NSTEMI, non-ST-elevation myocardial infarction, OAT, oral anticoagulation therapy; STEMI, ST-elevation myocardial infarction; WBC, white blood cells.

	ACTION [34]	CRUSADE [35]	ACUITY-HORIZONS [36]	PARIS [37]	PRECISE-DAPT [38]	BleeMACS [39]
Population	STEMI, NSTEMI	NSTEACS	ACS	Stable CAD, ACS	Stable CAD, ACS	ACS
Variables						
**Age**	**X**		**X**	**X**	**X**	**X**
Gender	X	X	X			
Heart rate	X	X				
Systolic BP or hypertension	X	X				X
**Hemoglobin**	**X**		**X**	**X**	**X**	**X**
Hematocrit		X				
WBC			X		X	
**Creatinine**	**X**	**X**	**X**	**X**	**X**	**X**
Diabetes	X	X				
Smoking				X		
Body mass *	X			X		
HF	X	X				
Vascular disease	X	X				X
Malignancy						X
OAT	X			X		
ECG changes	X					
ATT			X			
Type of ACS			X			
Prior bleeding					X	X
Bleeding outcome	In-hospital	In-hospital	30 days	2 years	12 months	12 months

**Table 3:** Randomized controlled trials comparing different durations of dual antiplatelet therapy. ASA, aspirin; BARC, Bleeding Academic Research Consortium; Mos, months; TIMI, Thrombolysis in Myocardial Infarction; ∨, statistically significant reduction in outcome in experimental group compared to control group; ∧, statistically significant increase in outcome in experimental group compared to control group; =, no statistical difference in outcome between the two groups.

Trial	N	Experimental Group	Mos	Control Group	Mos	Ischemic Outcome	Results	Bleeding Outcome	Results
CURE [3]	12,562	ASA + Clopidogrel	3–12	ASA + Placebo	3–12	Death from cardiovascular causes, non-fatal myocardial infarction, or stroke	∨	TIMI	∧
PLATO [9]	18,624	ASA + Ticagrelor	3–12	ASA + Clopidogrel	3–12	Death from vascular causes, myocardial infarction, or stroke	∨	TIMI	=
TRITON [8]	13,608	ASA + Prasugrel	6–15	ASA + Clopidogrel	6–15	Death from cardiovascular causes, non-fatal myocardial infarction, or non-fatal stroke	∨	TIMI	=
TWILIGHT [60]	7119	Ticagrelor + ASA Ticagrelor + Placebo	3 9	Ticagrelor + ASA	12	Death from any cause, non-fatal myocardial infarction, or non-fatal stroke	=	BARC 2, 3, 5	∨
GLOBAL LEADERS [61]	15,991	Ticagrelor + ASA Ticagrelor	1 23	Ticagrelor/Clopidogrel + ASA ASA	12 12	All-cause mortality or non-fatal myocardial infarction	=	BARC 3, 5	∨
TICO [62]	3056	Ticagrelor + ASA Ticagrelor	3 9	Ticagrelor + ASA	12	Death, myocardial infarction, stent thrombosis, stroke, and target vessel revascularization	=	TIMI	∨

**Table 4:** Definition and management of bleeding in patients on dual antiplatelet therapy (modified from European Guidelines [7]). DAPT, dual antiplatelet therapy; GI, gastrointestinal; GU, genitourinary; Hb, hemoglobin; i.v., intravenous; Plt, Platelets; PPI, proton pump inhibitor; RBC, red blood cell; SAPT, single antiplatelet therapy.

Severity Grade	Definition	Examples	DAPT Management	Other Recommendations
**Trivial**	Any bleeding not requiring medical intervention or further evaluation	Skin bruising, ecchymosis, self-resolving epistaxis, minimal conjunctival bleeding	Continue DAPT	Reassure the patient Identify preventive strategies Drug-adherence counselling
**Mild**	Any bleeding that requires medical attention without requiring hospitalization	Not self-resolving epistaxis, moderate conjunctival bleeding, GU or GI bleeding without significant blood loss, mild hemoptysis	Continue DAPT Consider shortening DAPT duration Consider DAPT de-escalation	Identify and treat bleeding-related conditions Add PPI if not present Drug adherence counselling
**Moderate**	Any bleeding associated with a significant blood loss (>3 g/dL Hb) and/or requiring hospitalization, hemodynamically stable and not evolving	GU, respiratory or GI bleeding with significant blood loss or requiring blood transfusion	Consider switching from DAPT to SAPT Reinitiate DAPT as soon as deemed safe Consider DAPT de-escalation Consider shortening DAPT duration	Identify and treat bleeding-related conditions i.v. PPI if GI bleeding Drug adherence counselling
**Severe**	Any bleeding requiring hospitalization, associated with a severe blood loss (>5 g/dL Hb), hemodynamically stable and not rapidly evolving	Severe GU, respiratory or GI bleeding.	Consider switching from DAPT to SAPT If bleeding persists despite treatment, consider stopping APT Re-evaluate need of APT once bleeding has ceased Consider shortening DAPT duration Consider DAPT de-escalation	RBC if Hb < 7–8 g/dL Consider Plt transfusion Urgent treatment of bleeding source if possible i.v. PPI if GI bleeding
**Life-threatening**	Any severe active bleeding putting patient’s life immediately at risk	Massive overt GU, respiratory or GI bleeding, active intracranial, spinal or intraocular hemorrhage, or any bleeding causing hemodynamic instability	Immediately discontinue all APT Re-evaluate need of APT once bleeding has ceased.	Fluid replacement RBC and Plt transfusion Urgent treatment of bleeding source if possible i.v. PPI if GI bleeding
